# The Complexity and Phylogenetic Continuity of Laughter and Smiles in Hominids

**DOI:** 10.3389/fpsyg.2021.648497

**Published:** 2021-06-03

**Authors:** Marina Davila-Ross, Guillaume Dezecache

**Affiliations:** ^1^Psychology Department, University of Portsmouth, Portsmouth, United Kingdom; ^2^Université Clermont Auvergne, CNRS, LAPSCO, Clermont-Ferrand, France

**Keywords:** laughter, smiles, laughing faces, open-mouth faces, evolution, principle of maximum parsimony

## Abstract

Laughter and smiles are often, but not always, associated with positive affect. These expressions of humans help to promote social relationships as well as the development of cognitive and socio-emotional skills and they may have a positive impact on health and well-being, hereby covering a selection of fitness-relevant benefits. Both laughter and smiles of positive affect also occur early in human development and across cultures, suggesting deep roots in human biology. The present work provides an evolutionary reconstruction of the evolution of human laughter and smiles of positive affect in form and function, based on the principle of maximum parsimony. According to the Complexity and Continuity Hypothesis, human laughter and smiles of positive affect must have evolved within the context of play from ancestral species. Furthermore, ancestral ape laughter and their open-mouth faces must already have been complex in form and function and changed over time via categorically different phylogenetic pathways to become characteristic, effective, and pervasive behaviors of everyday social interactions in humans.

## Introduction

Laughter and smiles of humans have often been discussed in close association with each other. Both play an important role in a wide range of daily social interactions (Owren and Bachorowski, 2003; Dezecache and Dunbar, 2012). They promote social cohesion (Provine, 2000; Dunbar and Mehu, 2008) as well as the development of cognitive and socio-emotional skills (Fredrickson, 2001; Gervais and Wilson, 2005) and they may even affect a person's health and well-being (Keltner and Bonanno, 1997; cf. Martin, 2002; Dunbar et al., 2012), all in all covering a selection of fitness-relevant benefits. Although it is important to be generally cautious when identifying emotional states of individuals based on their behavioral actions (Fridlund and Russell, 2006; Fridlund, 2014; Waller et al., 2017), laughter and smiles are clearly strongly linked to positive emotions in many situations and their corresponding neurochemical changes (Wild et al., 2003; Dunbar et al., 2012; Manninen et al., 2017). It is perhaps best observable as outbursts of affect in solitary contexts and in young children's play. Whereas, laughter and smiles often represent behavioral indicators of positive emotions in humans, which may serve in multiple ways, they can also be products of other emotions as well as functions (e.g., fear grins: van Hooff, 1972; Schadenfreude laughter: Szameitat et al., 2009a; for critical discussions on expressions and their relationships to emotions and communication, see Fridlund and Russell, 2006; Dezecache et al., 2013).

These expressions of positive affect seem to be deeply grounded in human biology. They occur in the first months of human development and continue to stay then mainly within positive contexts (Sroufe and Wunsch, 1972; Nwokah et al., 1994; Oller et al., 2013). Typically sounding laughter is even produced by those with no or close to no auditory experience, such as in congenitally deaf college students (Makagon et al., 2008). Furthermore, laughter and smiles occur in positive contexts across cultures, for instance in rough-and-tumble play of children (Grammer and Eibl-Eibesfeldt, 1990; Provine, 2000), and they are overall detected as positive expressions, albeit with cross-cultural differences in how they are more specifically processed (Masuda et al., 2008; Sauter et al., 2010; Bryant et al., 2016). Consequently, these positive expressions might reflect the more rudimentary, evolutionarily older forms of laughter and smiles and require special attention in the search for potential homologs in non-human primates (“primates” from here onwards).

It has long been noted that human and primate expressions of emotions may be similar in both anatomy and context, especially with regard to human laughter and smiles and the playful situations in which they may occur (Darwin, 1872; Andrew, 1963; Chevalier-Skolnikoff, 1973; Redican, 1982; Preuschoft, 1992). For instance, chimpanzee mothers may tickle their infants, who then produce play vocalizations and open-mouth faces (play faces), expressions they would also show during solitary play as well as play with peers, such as rough and tumble, tug of war, or play chase. Play vocalizations and open-mouth faces can be found among primates early in their development (Tomonaga et al., 2004; Bard et al., 2014) and across their different populations (e.g., in chimpanzees: Matsusaka, 2004; Davila-Ross et al., 2011). Interestingly, the way playing great apes produce their multimodal and unimodal expressions of play strongly resembles the way playing children produce laughter and open-mouth smiles of positive affect (Rothbart, 1973; Addyman et al., 2018), respectively. Whereas, such basic observations might naturally lead to the notion of phylogenetic continuity from primordial play expressions to human laughter and smiles of positive affect (Darwin, 1872; Redican, 1982), other possible explanations are that laughter and smiles are human-unique behaviors or that they evolved from different primordial expressions (van Hooff, 1972; Preuschoft and van Hooff, 1995).

In the last two decades, numerous in-depth studies on the form and function of primate play expressions were conducted that urge us to revisit the evolution of laughter and smiles. The goal of the present work is, thus, to examine these findings in combination with pioneering works on this topic in order to develop an evolutionary model of laughter and smiles, situated within the phylogeny of great apes and humans. Our evolutionary reconstruction from ancestral apes toward humans is mainly based on predictions we can make about the last common ancestor of extant great apes and humans, a relationship extracted from a multiplex phylogenetic clade that also includes other extinct species, which existed prior to the origin of modern humans. Because laughter is a multimodal expression that is primarily defined by its vocalization (Cosentino et al., 2016), we distinguish, when necessary in this work, between the terms “laugh vocalization” and “laughing face” to refer to its vocal and facial components, respectively (Ruch and Ekman, 2001; Drack et al., 2009). Smiles, in contrast, are the facial expressions that are not produced together with laugh vocalizations (Ekman et al., 1990; Iwase et al., 2002). Our evolutionary reconstruction is based on the principle of maximum parsimony. According to this principle, the most likely of alternative explanations on evolutionary pathways should involve the least number of predicted evolutionary steps for a given set of data (Saitou and Imanishi, 1989). It can be applied for any hard-wired multivariate traits (for primate expressions, see Geissmann, 2002; Davila-Ross and Geissmann, 2007; Davila-Ross et al., 2009).

## Discussion

### Play Vocalizations and Laughter

The play vocalizations of great apes, among the primates, have received much research attention because of the acoustic similarity with human laughter found in the closest evolutionary relatives of humans (Darwin, 1872; van Lawick-Goodall, 1968; Gervais and Wilson, 2005; Leavens, 2009). These ape vocalizations are often, but not always, occurring as a series of low-frequency staccato grunts, which can perhaps most readily be elicited by tickling (Vettin and Todt, 2005; Davila-Ross and Zimmermann, 2009; Provine, 2017). With the aim to test for such potential homologies and to situate the evolution of laugh vocalizations within the larger phylogenetic trajectory of the Hominidae, Davila-Ross et al. (2009, 2010) used raw acoustic data obtained from tickling-induced vocalizations of infant and juvenile great apes and human infants to conduct phylogenetic analyses. Their generated maximum-parsimony trees matched the phylogeny of extant great apes and humans that has been well-established by geneticists (Ruvolo et al., 1994; Wildman et al., 2002; McBrearty and Jablonski, 2005). This match and additional analyses, that revealed robustness of the tree topology, indicated a shared evolutionary origin (Davila-Ross et al., 2009). Because human infant laughter was included in this study, phylogenetic evidence was provided that human laughter evolved from ancestral apes within the context of play at least 10–16 million years ago (Davila-Ross et al., 2009, 2010).

These laugh vocalizations of great apes occur predominantly during their dyadic play (Davila-Ross and Zimmermann, 2009), where they seem to help prolong such playful encounters in chimpanzees (Matsusaka, 2004; Davila-Ross et al., 2011). Even when recorded laughter of conspecifics was played back in two previous studies, chimpanzees did not produce laughter outside of play (Berntson et al., 1989; Davila-Ross et al., 2014), suggesting a limitation in flexible use, in contrast to human laughter (see Provine, 1992). Pilot video playback tests involving orangutans, gorillas and chimpanzees provided no different results (Davila-Ross, personal observations).

Within the context of play, however, there seem to be notable differences in laughter among these primates that suggest a higher level of complexity in both form and function in the African great apes. Whereas, orangutans (the great apes evolutionarily most distanced from humans) produce spontaneous laughter during their playful encounters, it is rare (Davila-Ross and Zimmermann, 2009). Instead, they often emit play squeaks, another type of play vocalization (Davila-Ross et al., 2010). Compared to their Asian counterparts, African great apes (gorillas, chimpanzees, and bonobos) laugh frequently during social play (Vettin and Todt, 2005; Davila-Ross and Zimmermann, 2009), suggesting a higher level of signal relevance. In addition, chimpanzees may emit laugh responses to their playmates' laughter that are shorter than their spontaneously produced laughter (Davila-Ross et al., 2011).

Furthermore, orangutans produce laughter of an overall simpler spectral and temporal structure than the African apes. Orangutan laugh bouts are typically short in duration, with uniformly noisy calls of mostly consistently egressive airflow (Davila-Ross et al., 2009). The African apes, in contrast, produce laugh bouts that are longer in duration, with more calls that are produced quickly and with more vibration regime changes (Davila-Ross et al., 2009). They may laugh alternating between ingressive and egressive airflow (also described as play panting: Matsusaka, 2004) as well as with sustained, consistently egressive airflow (Davila-Ross et al., 2010), an ability that enables the continuous flow of speech in humans (Winkworth et al., 1995; MacLarnon and Hewitt, 1999). Some chimpanzees and bonobos were even heard to produce laugh bouts for minutes, which was possible via both airflow systems (Davila-Ross, personal observations).

Human and great ape laugh vocalizations seem to differ bioacoustically and perceptually primarily in regular voicing, airflow direction and vibration regimes (Davila-Ross et al., 2009). Voicing occurs when the vocal folds are vibrating with a high degree of regularity, leading to distinctive melodic sounds that mark human speech (Lieberman, 1975; cf. Owren et al., 1997). It is present in some human laugh episodes, for instance “Ha-ha” and “He-he” (Provine and Yong, 1991; Provine, 2000), but rarely in great apes (Vettin and Todt, 2005; Davila-Ross et al., 2009). Interestingly, unvoiced human laughter, which includes grunt-, snort- and song-like laugh episodes, is more common than voiced laughter (Bachorowski et al., 2001). Furthermore, human laughter shows primarily egressive airflow and a notably higher abundance of quickly produced vibration regimes than that of great apes, which contributes to their spectral complexity (Davila-Ross et al., 2009, 2010; for human laugh acoustics also see Bachorowski et al., 2001; Szameitat et al., 2009b).

### Open-Mouth Faces, Laughing Faces, and Smiles

Open-mouth faces of primates often occur during solitary play as well as social play and play invitations (Chevalier-Skolnikoff, 1974; Flack et al., 2004; Petru et al., 2009). Like play faces of mammals, in general, these primate expressions seem to guide play activities among the playmates by prolonging play and avoiding escalations into fights (Bekoff, 1995; Waller and Dunbar, 2005; Davila-Ross et al., 2011; Mancini et al., 2013). They can be spontaneously produced behaviors as well as responses to open-mouth faces of their playmates, for instance via rapid facial mimicry (Davila-Ross et al., 2008, 2011; Mancini et al., 2013; Palagi et al., 2019b).

**Figure 1:**
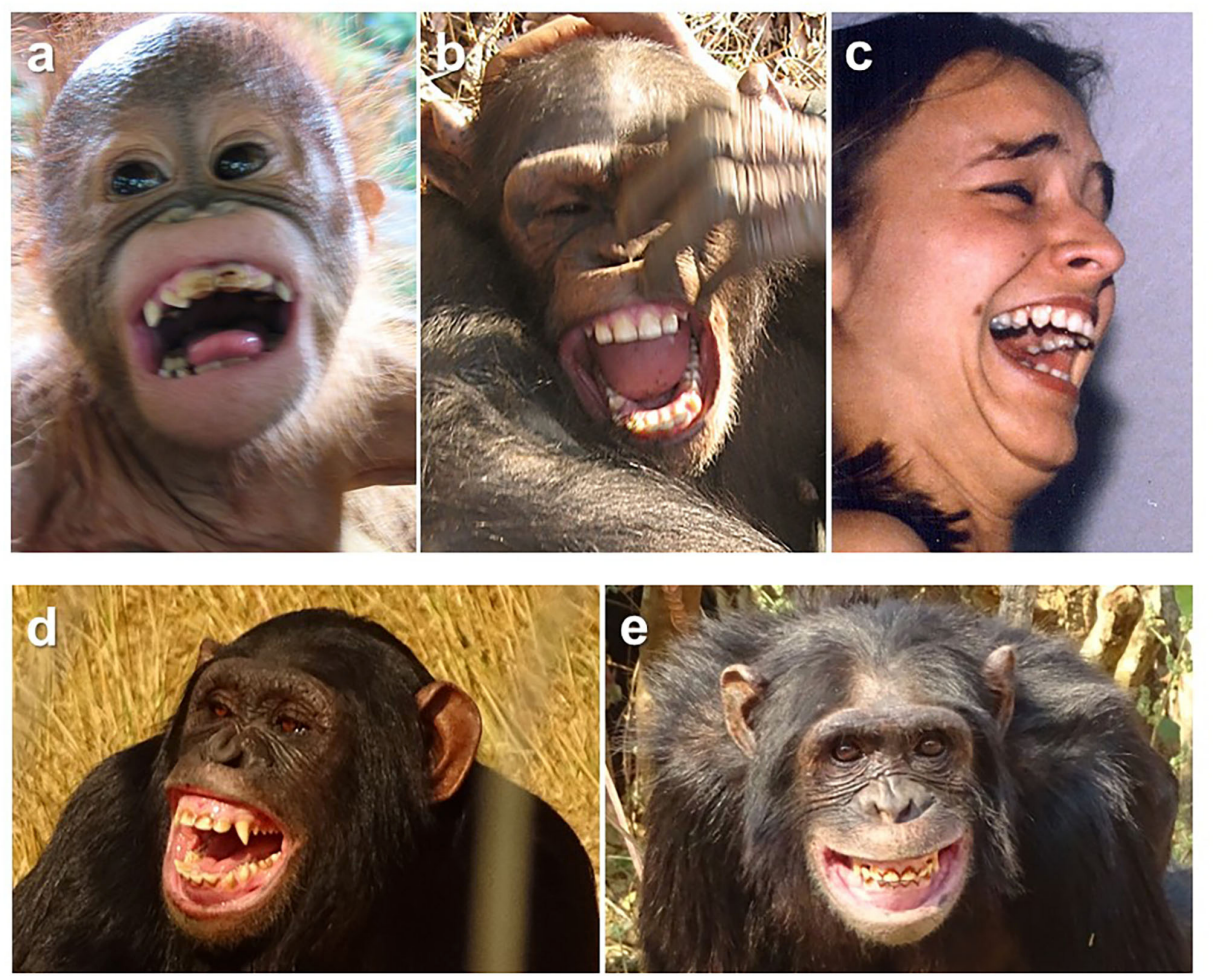
Primate and human facial expressions: **(a)** orangutan and **(b)** chimpanzee open-mouth faces and **(c)** human laughing face; **(d)** open-mouth and **(e)** closed-mouth silent bared-teeth displays of chimpanzees (two silent bared-teeth display pictures, credit: Helene Chotard).

In great ape play, open-mouth faces may be produced with laugh vocalizations as well as without them. Furthermore, these facial expressions show morphological commonalities with human laughing faces (see Figure 1) and smiles. Primate coding approaches that are based on the Facial Action Coding System (FACS: Ekman et al., 2002) provide special insight (for OrangFACS: Caeiro et al., 2013; for ChimpFACS: Vick et al., 2007). Such non-invasive methodologies allow researchers to systematically measure single facial movements of the underlying musculature shared by primates and humans to test for homologies (Ekman et al., 2002; Vick et al., 2007). Specifically, the open-mouth faces of play are marked by the contraction of the muscle *zygomaticus major*, which pulls the corners of the lips back and upwards, as well as by the opening of the lips (Parr et al., 2007; Davila-Ross et al., 2015; Waller et al., 2015), facial movements that characterize both laughing faces and smiles of humans (Ekman et al., 1990; Ruch and Ekman, 2001; Drack et al., 2009).

To test if human laughing faces and smiles emerged from pre-existing traits, Davila-Ross et al. (2015) measured with ChimpFACS the range of facial movements present in laughing chimpanzees during spontaneous play. The study revealed that laughing chimpanzees part their lips and pull them back as well as upwards while dropping their jaws; often they open their mouths further by raising their upper lips and sometimes they raise their cheeks, which causes crow's feet, besides showing other facial movements (Davila-Ross et al., 2015; see Figure 1). These movements matched those of laughing humans, which were identified by Drack et al. (2009) with FACS (also see Ekman et al., 1990; Ruch, 1993; Ruch and Ekman, 2001; Shiota et al., 2003). Moreover, the examined open-mouth faces of laughing chimpanzees included an almost identical set of facial configurations as the open-mouth faces without laughter (Davila-Ross et al., 2015), suggesting that they represent the same facial expressions. Therefore, based on the principle of maximum parsimony, the primordial open-mouth face of play represents the strongest candidate for a precursor of human laughing faces and smiles of positive affect—a phylogenetic reconstruction that involves no major evolutionary changes.

Indeed, the open-mouth face is the only primate expression that matches human laughing faces as well as human smiles in their close relationship to laughter, in addition to morphology and context. A different evolutionary model was provided by van Hooff (1972) in perhaps the most influential work on the evolution of human smiles over the past 50 years (see Lockard et al., 1977; Goldenthal et al., 1981; Preuschoft, 1992; Laidre and Yorzinski, 2005; Mehu and Dunbar, 2008), where he proposed another primate facial expression as the precursor of human smiles of positive affect, i.e., silent bared-teeth display (see Figure 1). According to van Hooff's (1972) Emancipation Hypothesis, the open-mouth variant of the silent bared-teeth display must have crossed contexts (from submissive to playful contexts) and morphologically converged with another facial display, i.e., open-mouth faces, as well as laugh vocalizations at a period following the last common ancestor of chimpanzees and humans in order to become smiles, emancipating then in function and crossing behavioral contexts in humans—a phylogenetic reconstruction which, like that of novel facial expressions, includes multiple major evolutionary changes.

It is important to note, however, that van Hooff (1972) also set the open-mouth face apart from other primate expressions as the most parsimonious explanation for a smile homolog, were it not for one then missing piece. Specifically, he pointed out that laughing children bare their upper tooth rows unlike the laughing chimpanzees he observed in Burgers' Zoo (van Hooff, 1972). Such open-mouth faces of chimpanzees and other primates are also referred to as relaxed open-mouth displays, a term that was used to indicate that during play the upper lip is relaxed, covering the upper teeth (van Hooff, 1972; Thierry et al., 1989). Over the last couple of decades, however, research demonstrated the exposure of upper teeth as part of the open-mouth face (also known as “relaxed open-mouth bared-teeth displays” and “full play faces”) in laughing great apes (Davila-Ross and Zimmermann, 2009; Davila-Ross et al., 2015; see Figure 1) and in playing primates in general (van Hooff and Preuschoft, 2003; Palagi, 2006; Waller and Cherry, 2012). This facial movement is primarily caused by the *levator labii superioris* muscle contraction which raises the upper lip toward the nose. Therefore, the rationale for silent bared-teeth displays does not seem to hold any longer when it comes to smile precursors of positive affect.

Clearly the facial variants of open-mouth faces of play are closely linked to each other. In chimpanzee social play, approximately half of the 17 identified open-mouth configurations of play seem to involve an exposure of the upper teeth, configurations that may change into one another within a single display event (Davila-Ross et al., 2015; Davila-Ross, personal observations). The open-mouth variants marked by the exposed and covered upper teeth are, thus, to some extent interchangeable behaviors and they regularly occur in both gentle play and rough-and-tumble (see Davila-Ross and Zimmermann, 2009), although the upper teeth tend to be exposed more often during the latter play type (Palagi, 2006; Waller and Cherry, 2012). Whereas, primates, in general, make use of both open-mouth variants during play (e.g., geladas: Mancini et al., 2013; Japanese macaques: Scopa and Palagi, 2016: orangutans: Davila-Ross and Zimmermann, 2009; gorillas: Waller and Cherry, 2012; bonobos: de Waal, 1988), some primate species differ in the predominant use of these variants (see Thierry et al., 1989; van Hooff and Preuschoft, 2003; Scopa and Palagi, 2016). According to the Power Asymmetry Hypothesis by Preuschoft and van Hooff (1997), it should be particularly important for primate species living in strict linear dominance hierarchies (e.g., pig-tailed macaques) to produce distinct signals that can be easily recognized by their conspecifics, consequently shrinking the chances of escalations into fights, compared to primate species of a notably more relaxed social system (e.g., Tonkean macaques). Consistent with this hypothesis, pig-tailed macaques, for example, often do not expose their upper tooth rows during play, making their play signals distinct from silent bared-teeth displays of other contexts, much in contrast to Tonkean macaques (Bobbitt et al., 1964; Thierry et al., 1989; see van Hooff and Preuschoft, 2003).

Without the upper teeth exposed, open-mouth faces have only been infrequently documented outside of play (Preuschoft, 1992; Waller and Dunbar, 2005). Interestingly, Jan van Hooff's observed such an open-mouth face by a chimpanzee named Mama, which occurred after a known researcher revealed himself behind a leopard mask (van Hooff and Preuschoft, 2003). We are also aware of a youtube video clip where a juvenile orangutan produced this display following a magic trick (accessed 1st of April 2021: https://www.youtube.com/watch?v=OLrYzY3jVPY&ab_channel=Hydrasound). A similarly unusual incident took place at the Serengeti Park Hodenhagen, where a juvenile female chimpanzee named Pia was play inviting her father by pulling his hair, a clearly incongruent but seemingly harmless event (Davila-Ross, personal observation). As her father would not budge, Pia left and went to a different island of the enclosure, laid down on the grass and seemed to relax for a while, until she all of a sudden started producing open-mouth faces (Davila-Ross, personal observation; see Supplementary Video). It seems reasonable to conclude that Pia's outburst was induced by a representation of the preceding incongruent playful encounter. Collectively, such rare incidents provide evidence that great apes are able to produce open-mouth faces outside of play after non-aggressive violations of expectations, similar to human infant smiles (Reddy, 1991; for a discussion on benign violations and humor, see McGraw and Warren, 2010; Eckert et al., 2020). With the upper teeth exposed, open-mouth faces of play and the silent-bared teeth displays show interesting similarities that are discussed in the next section.

### Complexity and Continuity Hypothesis of Laughter and Smiles

Empirical research on primate play expressions and human laughter and smiles of positive affect brings us back to the natural conclusion of phylogenetic continuity. Furthermore, with primordial open-mouth faces of play having evolved into human laughing faces and open-mouth smiles of positive affect, we can conclude that a shared ancestry of these two human facial expressions exists. Additional support comes from research on human facial morphology and physiology. Interestingly, human laughing faces and smiles of positive affect are both identified by *zygomaticus major* and include an overall similar configuration of facial muscle movements, such as *orbicularis oculi* muscle contractions (see Ekman et al., 1990; Shiota et al., 2003; Drack et al., 2009). Activations of *orbicularis oculi* raise the cheeks, causing the wrinkling around the eye corners, i.e., crow's feet, which characterizes Duchenne laughter/smiles, expressions arguably associated with felt positive emotions (Surakka and Hietanen, 1998; Ruch and Ekman, 2001; cf. Gunnery and Hall, 2015). In addition, a positron emission tomography (PET) scan study indicated that spontaneous laughter and smiles of positive affect, when produced by participants who watched funny videos, showed similar neural activations, predominantly in the bilateral supplementary motor area (SMA) and left putamen (Iwase et al., 2002).

Human laughter and smiles are also similar in function. Both may range from simple positive outbursts (Rothbart, 1973; Ekman et al., 1990) to highly complex behaviors, such as responses to humorous incidents and integral components in conversations (Owren and Bachorowski, 2003; Wild et al., 2003; Vettin and Todt, 2004; Arias et al., 2018). They promote cognitive and socio-emotional development (Fredrickson, 2001; Gervais and Wilson, 2005) and help to form, maintain and strengthen social relationships (Mehu et al., 2007; Dezecache and Dunbar, 2012; Wood and Niedenthal, 2018). Their positive effects may be further amplified when these expressions are shared among social partners (Provine, 1992; Hess and Bourgeois, 2010; cf. Dezecache et al., 2015) and when volitionally producing them (Bryant and Aktipis, 2014; Scott et al., 2014). Previously, human laughter and smiles have been identified as graded behaviors of intensity within positive contexts (Ekman, 1982; Redican, 1982; Bachorowski and Owren, 2001). According to the Diminutive Hypothesis, smiles have a lower arousal mode than laughter (Redican, 1982), a relationship that is also present in several languages (e.g., *rire* and *sourire* in French; *Lachen* and *Lächeln* in German). This hypothesis further implies that these two often interchangeable behaviors of positive contexts emerged from the same phylogenetic root (cf. Andrew, 1963; Redican, 1982), but it is hereby necessary to consider that laughter is predominantly a multimodal expression, unlike smiles. While human laugh faces are likely to be homologs of human smiles, empirical findings on primates suggest categorically different periods of evolutionary change for laugh vocalizations and smiles of positive affect. For laugh vocalizations, two main periods of evolutionary change among the hominids have been identified (Davila-Ross et al., 2010).

The first period of change for laugh vocalizations took place within great ape phylogeny. Laughter of the last common ancestor of extant great apes involved most likely a spontaneous, unvoiced vocalization of noise produced during social play, a vocalization that may have resembled a loud breathing (Davila-Ross et al., 2009). Laughter must have then become an increasingly complex and socially important vocalization, which was exchanged among playing conspecifics, as found in extant African apes (Davila-Ross et al., 2009). The second and predominant period of change for laugh vocalizations occurred closer to humans, after the divergence from a common ancestor with chimpanzees and bonobos. It must have been marked by regular voicing as well as consistently egressive airflow, two attributes of speech production (Davila-Ross et al., 2009). The increased presence of voicing in laughter may have heightened its level of perceived valence and arousal, as voiced laughter is processed as more positive in listeners than unvoiced laughter (Bachorowski and Owren, 2001). Interestingly, the main periods of change indicate categorically different phylogenetic pathways in laughter and smiles.

Specifically, open-mouth faces seem to have gone through one main period of evolutionary change in the past 10–16 million years. This facial expression of the last common ancestor of extant great apes was most likely already a behavior of high social relevance in play, with a complexity in both form and function, expressions that must have been used more flexibly than laughter (Davila-Ross and Zimmermann, 2009; Davila-Ross et al., 2015; Waller et al., 2015). Such complexity of open-mouth faces seems to be similarly present in monkeys (Mancini et al., 2013; Clark et al., 2020; see Preuschoft and van Hooff, 1995), perhaps even beyond primates (Palagi et al., 2019a; Taylor et al., 2019). Closer to humans and after the divergence from a common ancestor with chimpanzees and bonobos, these expressions may have involved more often *orbicularis oculi* muscle activations, possibly resulting in an increase in perceived valence and arousal (Ekman et al., 1990; Messinger et al., 2001; Soussignan, 2002; Davila-Ross et al., 2015), a change that is unrelated to the increase in voicing of laughter.

As part of the Complexity and Continuity Hypothesis, we therefore argue that great ape laughter and their open-mouth faces of play are homologs of the two arguably strongest behavioral indicators of positive affect in humans, expressions that are both frequently and, to some extent, similarly found in young children's play (Rothbart, 1973; Addyman et al., 2018). Consequently, humans are not unique in producing laughter and smiles of positively grounded motivations. Whereas, humans are known for having highly sophisticated social-cognitive abilities closely linked to cooperation unlike any other extant species (see Moll and Tomasello, 2007; Tomasello and Herrmann, 2010), these two important everyday expressions of social cohesion (Provine, 2000; Dunbar and Mehu, 2008) must have already existed on a pre-human basis, possibly to help initiate and prolong playful interactions with familiar conspecifics (Matsusaka, 2004; Waller and Dunbar, 2005; Davila-Ross et al., 2011; Mancini et al., 2013). Thus, the Complexity and Continuity Hypothesis contrasts the notion that human smiles of positive affect evolved within fear-related situations of ancestral species (see Emancipation Hypothesis: van Hooff, 1972; also see Andrew, 1963).

With the primate homologs of human laughter and smiles of positive affect identified based on the maximum parsimony principle, it is now important to critically evaluate how laughter and smiles became expressions of other motivations and functions (e.g., Schadenfreude: Szameitat et al., 2009a; mocking: Provine, 2000; embarrassed and polite smile: Ambadar et al., 2009). Regarding laugh vocalizations, it seems reasonable to conclude that such emancipation took place only after the last common ancestor of extant great apes and humans existed, because ape laughter is bioacoustically distinct (Davila-Ross et al., 2009; Taylor et al., 2021) and closely linked to play (Matsusaka, 2004; Davila-Ross et al., 2011), so that there cannot be variants with similar acoustic properties in other behavioral contexts. Furthermore, human infants within their first year of life produce various speech-related vocal types (protophones) free from contexts, but not laughter (Oller et al., 2013; for a comparative approach, see Dezecache et al., 2020). Consequently, laughter must have been used more flexibly closer toward humans, occurring in a wide range of everyday social interactions with gradually modifying acoustic properties (Owren and Bachorowski, 2003; Davila-Ross et al., 2010), perhaps accompanying key changes in language evolution (for laughter in conversation, see Vettin and Todt, 2004; Flamson and Bryant, 2013), when it also became an expression of other motivations and functions.

Regarding smiles of different motivations and functions, it is important to note that primates also expose their upper teeth when widely opening their mouths (e.g., silent-bared teeth displays and open-mouth threat faces) in contexts outside of play, namely in reconciliation, appeasement, affiliation, copulation and agonistic contexts (see Andrew, 1963; van Hooff, 1972; Weigel, 1979; Redican, 1982; Preuschoft, 1992; Liebal et al., 2004, 2006; Waller and Dunbar, 2005). As already mentioned in Jan van Hooff's (1972) important work on smile evolution, the silent bared-teeth displays show interesting similarities with open-mouth faces of play. More recently, FACS-based studies revealed that monkeys as well as apes may activate the same facial muscles across the two displays, although they seem to differ in their overall facial configurations (Parr and Waller, 2006; Parr et al., 2007; Davila-Ross et al., 2015; Waller et al., 2015; Clark et al., 2020) and perhaps in the motion pattern of muscle units, with the open-mouth faces of play showing the more dynamic pattern. Furthermore, these displays seem to instigate affiliative behaviors among interacting conspecifics across the behavioral contexts (Preuschoft, 1992; Bout and Thierry, 2005; Waller and Dunbar, 2005; de Marco and Visalberghi, 2007; Davila-Ross et al., 2011; Mancini et al., 2013).

Therefore, the possibility that open-mouth faces of play and silent bared-teeth displays are variants of each other should not be ignored, which means that the latter display might after all have had a role in smile evolution. In such case, however, its phylogenetic pathway would have been notably different from how it was presented in the Emancipation Hypothesis (see van Hooff, 1972; Preuschoft and van Hooff, 1995). Based on the data currently available, it is plausible that primordial forms of both open-mouth faces of play and silent bared-teeth displays emancipated in function, flexibly crossing behavioral contexts prior to the origin of hominids. This is further supported by data on the flexible use of primate facial expressions (Preuschoft, 1992; Waller and Dunbar, 2005; Davila-Ross et al., 2015; Scheider et al., 2016). An alternative explanation is that open-mouth faces of play are not related with silent bared-teeth displays, in which case the precursor of smiles of positive affect must have been used more freely across contexts after the last common ancestor of apes and humans existed, like laughter, to become a pervasive tool of human communication (see Owren and Bachorowski, 2003). More research is needed to test these two possible explanations. Both explanations, however, contrast with the Emancipation Hypothesis (van Hooff, 1972), where it was argued that fear-related displays emancipated in function closer toward human evolution.

In sum, the Complexity and Continuity Hypothesis of this work presents an evolutionary reconstruction of laughter and smiles of positive affect that reveals phylogenetic continuity. As evolution conserves hard-wired behavioral traits and their underlying processes rather than abolishes and rebuilds them, human laughter and smiles of positive affect must have evolved within the context of play in ancestral species. The Complexity and Continuity Hypothesis further states that their primordial displays must have already been complex in form and function ~10–16 million years ago and further changed over time via categorically different phylogenetic pathways to become characteristic, effective and pervasive behaviors of everyday human social interactions.

## Data Availability Statement

The original contributions presented in the study are included in the article/Supplementary Material, further inquiries can be directed to the corresponding authors.

## Ethics Statement

Written informed consent was obtained from the individual(s) for the publication of any potentially identifiable images or data included in this article.

## Author Contributions

MD-R and GD contributed to the write up. All authors contributed to the article and approved the submitted version.

## Conflict of Interest

The authors declare that the research was conducted in the absence of any commercial or financial relationships that could be construed as a potential conflict of interest.
